# 2,6-Bis(4*H*-1,2,4-triazol-4-yl)pyridine dihydrate

**DOI:** 10.1107/S1600536811041687

**Published:** 2011-10-12

**Authors:** De-quan Jiao, Xiao Tong Han, Qiong Zhou, Ying Wang

**Affiliations:** a Tianjin Key Laboratory of Structure and Performances of Functional Molecules, Tianjin Normal University, Tianjin 300387, People’s Republic of China

## Abstract

The asymmetric unit of the title compound, C_9_H_7_N_7_·2H_2_O, comprises three formula units. The dihedral angles between the triazole rings and the respective central pyridine rings are 4.87 (16)/1.39 (17), 6.46 (16)/7.61 (16) and 7.00 (16)/3.77 (17)°. The water mol­ecules form O—H⋯O hydrogen bonds between themselves and O—H⋯N hydrogen bonds with the N-atom acceptors of the triazole rings, producing a three-dimensional framework.

## Related literature

For the synthesis of the title compound, see: Wiley & Hart (1953 ▶). For properties of related compounds, see: Haasnoot (2000 ▶).
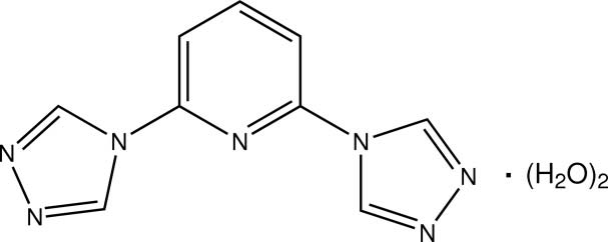


## Experimental

### 

#### Crystal data

C_9_H_7_N_7_·2H_2_O
*M* *_r_* = 249.25
Monoclinic, 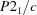

*a* = 9.7211 (17) Å
*b* = 17.921 (3) Å
*c* = 19.603 (4) Å
β = 91.333 (3)°
*V* = 3414.2 (10) Å^3^
*Z* = 12
Mo *K*α radiation
μ = 0.11 mm^−1^
*T* = 566 K
0.32 × 0.16 × 0.08 mm

#### Data collection

Bruker SMART CCD area-detector diffractometer
Absorption correction: multi-scan (*SADABS*; Sheldrick, 1996 ▶) *T*_min_ = 0.966, *T*_max_ = 0.991
19614 measured reflections
6030 independent reflections
3151 reflections with *I* > 2σ(*I*)
*R*_int_ = 0.057

#### Refinement

*R*[*F* ^2^ > 2σ(*F* ^2^)] = 0.051
*wR*(*F* ^2^) = 0.133
*S* = 1.00
6030 reflections
523 parameters
18 restraints
H atoms treated by a mixture of independent and constrained refinement
Δρ_max_ = 0.17 e Å^−3^
Δρ_min_ = −0.25 e Å^−3^

### 

Data collection: *SMART* (Bruker, 2008 ▶); cell refinement: *SAINT* (Bruker, 2008 ▶); data reduction: *SAINT*; program(s) used to solve structure: *SHELXS97* (Sheldrick, 2008 ▶); program(s) used to refine structure: *SHELXL97* (Sheldrick, 2008 ▶); molecular graphics: *SHELXTL* (Sheldrick, 2008 ▶); software used to prepare material for publication: *SHELXTL*.

## Supplementary Material

Crystal structure: contains datablock(s) I, global. DOI: 10.1107/S1600536811041687/go2026sup1.cif

Structure factors: contains datablock(s) I. DOI: 10.1107/S1600536811041687/go2026Isup2.hkl

Supplementary material file. DOI: 10.1107/S1600536811041687/go2026Isup3.cml

Additional supplementary materials:  crystallographic information; 3D view; checkCIF report

## Figures and Tables

**Table 1:** Hydrogen-bond geometry (Å, °)

*D*—H⋯*A*	*D*—H	H⋯*A*	*D*⋯*A*	*D*—H⋯*A*
O6—H6*A*⋯N21^i^	0.86 (1)	1.95 (1)	2.801 (3)	172 (3)
O6—H6*B*⋯N1^ii^	0.86 (1)	2.04 (1)	2.878 (3)	166 (3)
O5—H5*A*⋯O6	0.87 (1)	1.86 (2)	2.707 (3)	166 (4)
O5—H5*B*⋯O1^iii^	0.86 (1)	2.01 (1)	2.864 (4)	171 (4)
O4—H4*A*⋯N14^iv^	0.86 (1)	1.95 (1)	2.808 (3)	173 (4)
O4—H4*B*⋯N8^v^	0.86 (1)	2.09 (2)	2.907 (3)	160 (3)
O3—H3*B*⋯O5	0.86 (1)	2.03 (2)	2.868 (4)	166 (4)
O3—H3*A*⋯O4	0.85 (1)	2.01 (1)	2.855 (3)	173 (3)
O2—H2*B*⋯O4	0.86 (1)	2.00 (1)	2.851 (4)	172 (4)
O2—H2*A*⋯O1	0.87 (1)	2.03 (1)	2.886 (3)	171 (3)
O1—H1*B*⋯N15^vi^	0.86 (1)	2.05 (2)	2.861 (3)	158 (3)
O1—H1*A*⋯N7	0.86 (1)	1.97 (1)	2.820 (3)	171 (3)

Symmetry codes: (i) 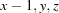; (ii) 
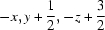
; (iii) 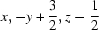; (iv) 
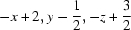
; (v) 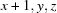; (vi) 
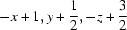
.

**Fig. 1.**
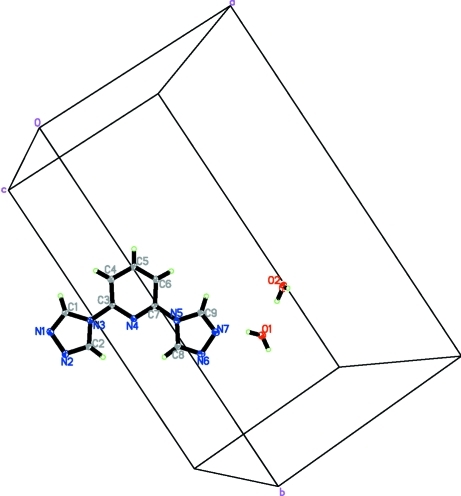
View of the molecule of (I) showing the atom-labelling scheme. Displacement ellipsoids are drawn at the 50% probability level.

**Fig. 2.**
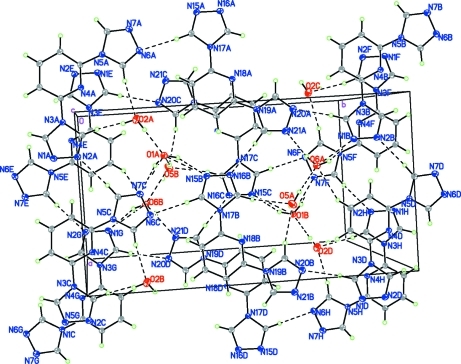
The crystal packing for (I), with the H bond interactions shown as dashed lines.

## supplementary crystallographic information

### Comment

Many molecular-based compounds exhibit interesting magnetic and luminescent properties, (Haasnoot, 2000). One of the requirements for producing such macroscopic properties is to create interactions between the molecular units and the active sites within the crystal lattices. 1,2,4-triazole and, in particular, its derivatives are interesting bridging ligands.

The asymmetric unit of (I) comprises three C_9_H_7_N_7_.2H_2_O components of those ilustrated in Figure 1. The dihedral angles between the triazole rings and the respective central pyridine rings are (atoms named indicate the relevant rings) N3/N4 4.87 (16)°, N4/N5 1.39 (17)°, N10/N11 6.46 (16)°, N11/N12 7.61 (16)°, N17/N18 7.00 (16)° and N18/N19 3.77 (17)°. The water molecules form O—H···O hydrogen bonds between themselves and O—H···N bonds with the N-atom acceptors of the triazole rings, producing a three-dimensional framework. (Table 1, Figure 2).

### Experimental

A mixture of 1.3 g (0.012 mol) of 2,6-diaminopyridine and 2.0 g (0.023 mol) of diformylhydrazine was heated slowly to 160–170 °C for 30 min. The crystals, which separated on cooling, were collected and recrystallized form water and acetonitrile to give 0.8 g of (I) (yield 20%). After several recrystallizations from water, the air-dried product was obtained as white needles, m.p. 325–327 K (placed in hot bolck at 320 K). The analysis was obtained on the air-dried sample. Anal. Calc. for C_9_H_11_N_7_O_2_ (%): C, 46.75; H, 3.92. Found (%): C, 46.89; H, 4.00.

### Refinement

Positional parameters of all the H atoms were calculated geometrically and were allowed to ride on the C or O atoms with C—H = 0.93 Å and O—H = 0.85 Å and *U*iso(H) = 1.2 or 1.5 times *U*eq (C or O). The hydrogen atoms of the water molecules were located from difference maps and refined with isotropic temperature factors.In the case of atoms O2 and O3 the angle between the H atoms was restained to be a value close to 104°.

### Crystal data

**Table:**

C_9_H_7_N_7_·2H_2_O	*F*(000) = 1560
*M**_r_* = 249.25	*D*_x_ = 1.455 Mg m^−^^3^
Monoclinic, *P*2_1_/*c*	Mo *K*α radiation, λ = 0.71073 Å
*a* = 9.7211 (17) Å	Cell parameters from 1505 reflections
*b* = 17.921 (3) Å	θ = 2.4–21.4°
*c* = 19.603 (4) Å	µ = 0.11 mm^−^^1^
β = 91.333 (3)°	*T* = 566 K
*V* = 3414.2 (10) Å^3^	PLATE, colorless
*Z* = 12	0.32 × 0.16 × 0.08 mm

### Data collection

**Table:**

Bruker SMART CCD area-detector diffractometer	6030 independent reflections
Radiation source: fine-focus sealed tube	3151 reflections with *I* > 2σ(*I*)
graphite	*R*_int_ = 0.057
φ and ω scans	θ_max_ = 25.0°, θ_min_ = 2.1°
Absorption correction: multi-scan (*SADABS*; Sheldrick, 1996)	*h* = −11→11
*T*_min_ = 0.966, *T*_max_ = 0.991	*k* = −21→21
19614 measured reflections	*l* = −23→18

### Refinement

**Table:**

Refinement on *F*^2^	Primary atom site location: structure-invariant direct methods
Least-squares matrix: full	Secondary atom site location: difference Fourier map
*R*[*F*^2^ > 2σ(*F*^2^)] = 0.051	Hydrogen site location: inferred from neighbouring sites
*wR*(*F*^2^) = 0.133	H atoms treated by a mixture of independent and constrained refinement
*S* = 1.00	*w* = 1/[σ^2^(*F*_o_^2^) + (0.0378*P*)^2^ + 0.9562*P*] where *P* = (*F*_o_^2^ + 2*F*_c_^2^)/3
6030 reflections	(Δ/σ)_max_ < 0.001
523 parameters	Δρ_max_ = 0.17 e Å^−^^3^
18 restraints	Δρ_min_ = −0.25 e Å^−^^3^

### Special details

**Table:**

Geometry. All e.s.d.'s (except the e.s.d. in the dihedral angle between two l.s. planes) are estimated using the full covariance matrix. The cell e.s.d.'s are taken into account individually in the estimation of e.s.d.'s in distances, angles and torsion angles; correlations between e.s.d.'s in cell parameters are only used when they are defined by crystal symmetry. An approximate (isotropic) treatment of cell e.s.d.'s is used for estimating e.s.d.'s involving l.s. planes.
Refinement. Refinement of *F*^2^ against ALL reflections. The weighted *R*-factor *wR* and goodness of fit *S* are based on *F*^2^, conventional *R*-factors *R* are based on *F*, with *F* set to zero for negative *F*^2^. The threshold expression of *F*^2^ > σ(*F*^2^) is used only for calculating *R*-factors(gt) *etc*. and is not relevant to the choice of reflections for refinement. *R*-factors based on *F*^2^ are statistically about twice as large as those based on *F*, and *R*- factors based on ALL data will be even larger.

### Fractional atomic coordinates and isotropic or equivalent isotropic displacement parameters (Å^2^)

**Table:**

	*x*	*y*	*z*	*U*_iso_*/*U*_eq_	
O1	0.7154 (2)	0.78220 (11)	0.96334 (15)	0.0661 (7)	
H1A	0.654 (3)	0.7512 (12)	0.9490 (19)	0.099*	
H1B	0.694 (3)	0.8260 (8)	0.9486 (19)	0.099*	
O2	0.9471 (3)	0.69444 (15)	0.92087 (15)	0.0823 (8)	
H2A	0.879 (3)	0.719 (2)	0.9382 (16)	0.123*	
H2B	0.943 (4)	0.701 (2)	0.8775 (6)	0.123*	
O3	0.7061 (3)	0.61811 (12)	0.72477 (16)	0.0778 (8)	
H3A	0.777 (3)	0.6395 (18)	0.7419 (18)	0.117*	
H3B	0.673 (4)	0.6445 (18)	0.6918 (15)	0.117*	
O4	0.9378 (2)	0.70073 (12)	0.77552 (15)	0.0677 (7)	
H4A	1.004 (2)	0.6707 (13)	0.7666 (19)	0.102*	
H4B	0.960 (3)	0.7445 (8)	0.762 (2)	0.102*	
O5	0.6322 (3)	0.69516 (15)	0.60102 (15)	0.0821 (8)	
H5A	0.558 (3)	0.719 (2)	0.6101 (18)	0.123*	
H5B	0.648 (4)	0.701 (2)	0.5583 (8)	0.123*	
O6	0.4062 (2)	0.78247 (12)	0.60995 (15)	0.0728 (8)	
H6A	0.341 (3)	0.7504 (13)	0.606 (2)	0.109*	
H6B	0.375 (3)	0.8260 (8)	0.600 (2)	0.109*	
N1	−0.2572 (2)	0.41842 (13)	0.91734 (13)	0.0467 (7)	
N2	−0.2565 (2)	0.49602 (13)	0.91358 (13)	0.0471 (7)	
N3	−0.0433 (2)	0.45570 (11)	0.91796 (12)	0.0364 (6)	
N4	0.1588 (2)	0.52262 (11)	0.91983 (11)	0.0339 (6)	
N5	0.3497 (2)	0.59867 (12)	0.92021 (12)	0.0386 (6)	
N6	0.3602 (3)	0.72068 (13)	0.92221 (15)	0.0541 (8)	
N7	0.4924 (2)	0.69199 (13)	0.91984 (15)	0.0578 (8)	
N8	0.0804 (2)	0.83897 (12)	0.74510 (14)	0.0474 (7)	
N9	0.0826 (2)	0.91653 (12)	0.74574 (13)	0.0448 (7)	
N10	0.2947 (2)	0.87465 (11)	0.75369 (12)	0.0352 (6)	
N11	0.4984 (2)	0.94024 (11)	0.75457 (12)	0.0337 (6)	
N12	0.6900 (2)	1.01533 (11)	0.75413 (12)	0.0362 (6)	
N13	0.7018 (2)	1.13674 (13)	0.74646 (13)	0.0490 (7)	
N14	0.8339 (2)	1.10798 (13)	0.74444 (13)	0.0477 (7)	
N15	0.4265 (2)	0.41749 (12)	0.57083 (13)	0.0453 (7)	
N16	0.4285 (2)	0.49419 (12)	0.58098 (13)	0.0444 (7)	
N17	0.6411 (2)	0.45391 (11)	0.57956 (12)	0.0339 (6)	
N18	0.8443 (2)	0.52004 (12)	0.58430 (12)	0.0355 (6)	
N19	1.0366 (2)	0.59472 (12)	0.58859 (12)	0.0391 (6)	
N20	1.0484 (3)	0.71698 (13)	0.58745 (16)	0.0625 (8)	
N21	1.1808 (3)	0.68779 (14)	0.58657 (14)	0.0560 (8)	
C1	−0.1310 (3)	0.39664 (15)	0.92001 (16)	0.0433 (8)	
H1	−0.1034	0.3470	0.9230	0.052*	
C2	−0.1282 (3)	0.51609 (15)	0.91468 (15)	0.0419 (8)	
H2	−0.0981	0.5653	0.9134	0.050*	
C3	0.1020 (3)	0.45514 (14)	0.91807 (15)	0.0352 (7)	
C4	0.1745 (3)	0.38948 (15)	0.91611 (16)	0.0484 (9)	
H4	0.1304	0.3434	0.9150	0.058*	
C5	0.3165 (3)	0.39510 (16)	0.91587 (18)	0.0543 (9)	
H5	0.3698	0.3521	0.9145	0.065*	
C6	0.3790 (3)	0.46336 (14)	0.91766 (16)	0.0470 (9)	
H6	0.4743	0.4679	0.9178	0.056*	
C7	0.2948 (3)	0.52521 (14)	0.91926 (14)	0.0344 (7)	
C8	0.2778 (3)	0.66368 (15)	0.92221 (16)	0.0462 (8)	
H8	0.1824	0.6669	0.9234	0.055*	
C9	0.4838 (3)	0.61994 (16)	0.91853 (16)	0.0486 (9)	
H9	0.5583	0.5875	0.9167	0.058*	
C10	0.2067 (3)	0.81618 (15)	0.74954 (16)	0.0457 (8)	
H10	0.2334	0.7663	0.7498	0.055*	
C11	0.2113 (3)	0.93586 (15)	0.75085 (14)	0.0398 (7)	
H11	0.2424	0.9849	0.7524	0.048*	
C12	0.4406 (2)	0.87350 (14)	0.75887 (14)	0.0334 (7)	
C13	0.5117 (3)	0.80762 (15)	0.76775 (16)	0.0452 (8)	
H13	0.4666	0.7620	0.7705	0.054*	
C14	0.6524 (3)	0.81262 (15)	0.77231 (17)	0.0488 (9)	
H14	0.7046	0.7696	0.7787	0.059*	
C15	0.7168 (3)	0.88044 (15)	0.76760 (16)	0.0430 (8)	
H15	0.8122	0.8845	0.7698	0.052*	
C16	0.6338 (3)	0.94236 (14)	0.75953 (14)	0.0347 (7)	
C17	0.6190 (3)	1.08051 (15)	0.75224 (16)	0.0437 (8)	
H17	0.5238	1.0843	0.7548	0.052*	
C18	0.8236 (3)	1.03616 (15)	0.74905 (15)	0.0426 (8)	
H18	0.8976	1.0033	0.7489	0.051*	
C19	0.5535 (3)	0.39572 (15)	0.57048 (16)	0.0436 (8)	
H19	0.5810	0.3465	0.5647	0.052*	
C20	0.5561 (3)	0.51389 (15)	0.58591 (15)	0.0408 (8)	
H20	0.5861	0.5626	0.5929	0.049*	
C21	0.7878 (3)	0.45277 (14)	0.58206 (14)	0.0326 (7)	
C22	0.8581 (3)	0.38678 (15)	0.58217 (15)	0.0401 (8)	
H22	0.8129	0.3411	0.5804	0.048*	
C23	0.9995 (3)	0.39140 (15)	0.58510 (16)	0.0443 (8)	
H23	1.0520	0.3480	0.5854	0.053*	
C24	1.0638 (3)	0.45981 (15)	0.58760 (15)	0.0413 (8)	
H24	1.1592	0.4639	0.5896	0.050*	
C25	0.9809 (3)	0.52181 (14)	0.58709 (15)	0.0358 (7)	
C26	0.9662 (3)	0.66025 (16)	0.58895 (18)	0.0559 (10)	
H26	0.8709	0.6639	0.5901	0.067*	
C27	1.1704 (3)	0.61615 (16)	0.58718 (16)	0.0482 (8)	
H27	1.2446	0.5835	0.5867	0.058*	

### Atomic displacement parameters (Å^2^)

**Table:**

	*U*^11^	*U*^22^	*U*^33^	*U*^12^	*U*^13^	*U*^23^
O1	0.0398 (13)	0.0492 (13)	0.109 (2)	0.0011 (10)	−0.0098 (13)	−0.0046 (14)
O2	0.0740 (18)	0.0638 (16)	0.109 (2)	0.0206 (13)	0.0131 (17)	0.0014 (18)
O3	0.0645 (17)	0.0626 (16)	0.106 (3)	−0.0161 (13)	−0.0046 (15)	0.0018 (15)
O4	0.0360 (13)	0.0498 (13)	0.118 (2)	−0.0005 (10)	0.0105 (13)	−0.0040 (15)
O5	0.0698 (18)	0.0691 (17)	0.107 (2)	0.0234 (14)	0.0024 (16)	0.0062 (18)
O6	0.0393 (13)	0.0498 (13)	0.129 (2)	0.0028 (10)	−0.0090 (14)	−0.0066 (16)
N1	0.0353 (15)	0.0441 (15)	0.061 (2)	−0.0023 (12)	0.0029 (13)	−0.0056 (13)
N2	0.0373 (15)	0.0420 (15)	0.062 (2)	0.0040 (11)	−0.0004 (13)	−0.0034 (13)
N3	0.0310 (13)	0.0298 (12)	0.0484 (18)	0.0015 (11)	0.0026 (11)	−0.0044 (11)
N4	0.0298 (13)	0.0309 (12)	0.0412 (17)	−0.0004 (10)	0.0012 (11)	0.0022 (11)
N5	0.0317 (13)	0.0319 (13)	0.0520 (18)	−0.0016 (10)	−0.0013 (12)	0.0042 (11)
N6	0.0444 (16)	0.0377 (14)	0.080 (2)	−0.0068 (12)	0.0027 (14)	0.0004 (14)
N7	0.0389 (16)	0.0442 (16)	0.090 (2)	−0.0045 (12)	0.0004 (15)	0.0004 (15)
N8	0.0336 (14)	0.0402 (14)	0.068 (2)	−0.0001 (11)	0.0041 (13)	−0.0020 (13)
N9	0.0315 (14)	0.0442 (15)	0.0587 (19)	0.0024 (11)	0.0030 (12)	0.0008 (13)
N10	0.0274 (12)	0.0296 (12)	0.0487 (17)	0.0001 (10)	0.0022 (11)	−0.0013 (11)
N11	0.0267 (12)	0.0322 (12)	0.0421 (16)	0.0026 (10)	−0.0008 (10)	−0.0003 (11)
N12	0.0276 (12)	0.0301 (12)	0.0509 (18)	−0.0025 (10)	0.0005 (11)	−0.0002 (12)
N13	0.0429 (15)	0.0376 (14)	0.067 (2)	−0.0027 (12)	0.0027 (13)	0.0070 (13)
N14	0.0407 (15)	0.0441 (15)	0.058 (2)	−0.0089 (12)	0.0003 (13)	0.0015 (13)
N15	0.0320 (14)	0.0385 (14)	0.065 (2)	−0.0043 (11)	−0.0005 (13)	0.0064 (13)
N16	0.0321 (14)	0.0437 (15)	0.058 (2)	0.0009 (11)	0.0023 (12)	0.0012 (13)
N17	0.0287 (12)	0.0302 (12)	0.0429 (17)	−0.0002 (10)	0.0000 (11)	0.0018 (11)
N18	0.0299 (13)	0.0345 (13)	0.0423 (17)	0.0015 (10)	0.0019 (11)	0.0015 (11)
N19	0.0303 (13)	0.0348 (13)	0.0522 (18)	−0.0024 (11)	−0.0019 (12)	−0.0010 (12)
N20	0.0495 (17)	0.0432 (16)	0.095 (3)	−0.0043 (14)	0.0004 (16)	−0.0010 (15)
N21	0.0435 (16)	0.0473 (16)	0.077 (2)	−0.0086 (13)	−0.0016 (14)	0.0052 (15)
C1	0.0402 (17)	0.0327 (16)	0.057 (2)	−0.0018 (14)	0.0015 (15)	−0.0022 (15)
C2	0.0384 (17)	0.0344 (16)	0.053 (2)	0.0030 (14)	−0.0020 (15)	−0.0015 (15)
C3	0.0323 (16)	0.0302 (15)	0.043 (2)	−0.0007 (12)	0.0024 (14)	0.0019 (13)
C4	0.0411 (18)	0.0290 (16)	0.075 (3)	0.0002 (13)	0.0042 (17)	−0.0064 (15)
C5	0.0389 (18)	0.0368 (17)	0.087 (3)	0.0104 (14)	0.0042 (17)	−0.0047 (17)
C6	0.0314 (16)	0.0366 (17)	0.073 (3)	0.0040 (13)	0.0050 (16)	−0.0012 (16)
C7	0.0360 (16)	0.0276 (14)	0.039 (2)	−0.0016 (12)	0.0019 (14)	0.0005 (13)
C8	0.0403 (18)	0.0357 (17)	0.063 (2)	0.0020 (14)	−0.0007 (16)	0.0036 (16)
C9	0.0364 (17)	0.0409 (18)	0.068 (3)	−0.0007 (14)	−0.0032 (16)	0.0034 (16)
C10	0.0327 (17)	0.0329 (16)	0.072 (3)	0.0004 (13)	0.0010 (16)	−0.0039 (15)
C11	0.0348 (17)	0.0376 (16)	0.047 (2)	0.0049 (13)	0.0031 (14)	0.0011 (14)
C12	0.0251 (14)	0.0335 (15)	0.041 (2)	0.0015 (12)	−0.0003 (13)	−0.0058 (13)
C13	0.0380 (17)	0.0311 (15)	0.066 (2)	0.0011 (13)	−0.0020 (16)	−0.0004 (15)
C14	0.0388 (18)	0.0360 (17)	0.071 (3)	0.0123 (13)	−0.0063 (16)	−0.0029 (16)
C15	0.0273 (15)	0.0428 (17)	0.059 (2)	0.0034 (13)	−0.0029 (14)	−0.0045 (16)
C16	0.0302 (16)	0.0332 (15)	0.041 (2)	0.0008 (12)	−0.0011 (13)	−0.0064 (14)
C17	0.0350 (16)	0.0382 (17)	0.058 (2)	0.0014 (14)	0.0019 (15)	0.0013 (15)
C18	0.0334 (17)	0.0452 (18)	0.049 (2)	−0.0012 (14)	0.0000 (15)	−0.0014 (16)
C19	0.0383 (17)	0.0314 (15)	0.061 (2)	−0.0043 (13)	−0.0044 (15)	−0.0010 (15)
C20	0.0377 (17)	0.0342 (16)	0.051 (2)	0.0031 (14)	0.0024 (15)	−0.0023 (15)
C21	0.0273 (15)	0.0339 (15)	0.0368 (19)	−0.0003 (12)	0.0017 (13)	0.0016 (13)
C22	0.0384 (17)	0.0305 (15)	0.051 (2)	−0.0015 (13)	0.0012 (15)	−0.0026 (14)
C23	0.0401 (18)	0.0321 (16)	0.061 (2)	0.0073 (13)	0.0020 (16)	−0.0006 (15)
C24	0.0238 (15)	0.0435 (17)	0.057 (2)	0.0048 (13)	−0.0017 (14)	−0.0008 (15)
C25	0.0342 (16)	0.0325 (15)	0.041 (2)	−0.0018 (13)	0.0011 (14)	−0.0011 (14)
C26	0.0396 (18)	0.0373 (18)	0.091 (3)	−0.0021 (15)	−0.0006 (18)	0.0027 (18)
C27	0.0360 (18)	0.0460 (19)	0.062 (2)	−0.0037 (14)	−0.0041 (16)	0.0043 (16)

### Geometric parameters (Å, °)

**Table:**

O1—H1A	0.857 (10)	N17—C20	1.363 (3)
O1—H1B	0.859 (10)	N17—C21	1.425 (3)
O2—H2A	0.868 (10)	N18—C21	1.325 (3)
O2—H2B	0.858 (10)	N18—C25	1.329 (3)
O3—H3A	0.854 (10)	N19—C27	1.357 (3)
O3—H3B	0.856 (10)	N19—C26	1.359 (3)
O4—H4A	0.861 (10)	N19—C25	1.414 (3)
O4—H4B	0.856 (10)	N20—C26	1.294 (3)
O5—H5A	0.868 (10)	N20—N21	1.390 (3)
O5—H5B	0.861 (10)	N21—C27	1.288 (3)
O6—H6A	0.861 (10)	C1—H1	0.9300
O6—H6B	0.858 (10)	C2—H2	0.9300
N1—C1	1.288 (3)	C3—C4	1.373 (3)
N1—N2	1.393 (3)	C4—C5	1.384 (4)
N2—C2	1.298 (3)	C4—H4	0.9300
N3—C1	1.360 (3)	C5—C6	1.366 (4)
N3—C2	1.361 (3)	C5—H5	0.9300
N3—C3	1.413 (3)	C6—C7	1.379 (3)
N4—C7	1.323 (3)	C6—H6	0.9300
N4—C3	1.329 (3)	C8—H8	0.9300
N5—C9	1.359 (3)	C9—H9	0.9300
N5—C8	1.359 (3)	C10—H10	0.9300
N5—C7	1.421 (3)	C11—H11	0.9300
N6—C8	1.298 (3)	C12—C13	1.377 (3)
N6—N7	1.386 (3)	C13—C14	1.371 (4)
N7—C9	1.294 (3)	C13—H13	0.9300
N8—C10	1.295 (3)	C14—C15	1.371 (4)
N8—N9	1.390 (3)	C14—H14	0.9300
N9—C11	1.300 (3)	C15—C16	1.379 (3)
N10—C10	1.354 (3)	C15—H15	0.9300
N10—C11	1.364 (3)	C17—H17	0.9300
N10—C12	1.420 (3)	C18—H18	0.9300
N11—C16	1.318 (3)	C19—H19	0.9300
N11—C12	1.325 (3)	C20—H20	0.9300
N12—C18	1.357 (3)	C21—C22	1.366 (3)
N12—C17	1.357 (3)	C22—C23	1.377 (4)
N12—C16	1.422 (3)	C22—H22	0.9300
N13—C17	1.296 (3)	C23—C24	1.376 (4)
N13—N14	1.385 (3)	C23—H23	0.9300
N14—C18	1.294 (3)	C24—C25	1.373 (3)
N15—C19	1.295 (3)	C24—H24	0.9300
N15—N16	1.389 (3)	C26—H26	0.9300
N16—C20	1.291 (3)	C27—H27	0.9300
N17—C19	1.356 (3)		
			
H1A—O1—H1B	108.9 (16)	N6—C8—N5	110.9 (3)
H2A—O2—H2B	107.5 (16)	N6—C8—H8	124.5
H3A—O3—H3B	109.4 (16)	N5—C8—H8	124.5
H4A—O4—H4B	108.5 (16)	N7—C9—N5	109.9 (2)
H5A—O5—H5B	107.8 (16)	N7—C9—H9	125.0
H6A—O6—H6B	109.3 (16)	N5—C9—H9	125.0
C1—N1—N2	107.4 (2)	N8—C10—N10	110.9 (2)
C2—N2—N1	106.4 (2)	N8—C10—H10	124.6
C1—N3—C2	103.9 (2)	N10—C10—H10	124.6
C1—N3—C3	128.4 (2)	N9—C11—N10	111.0 (2)
C2—N3—C3	127.6 (2)	N9—C11—H11	124.5
C7—N4—C3	116.5 (2)	N10—C11—H11	124.5
C9—N5—C8	104.7 (2)	N11—C12—C13	124.7 (2)
C9—N5—C7	128.3 (2)	N11—C12—N10	114.0 (2)
C8—N5—C7	127.0 (2)	C13—C12—N10	121.3 (2)
C8—N6—N7	106.3 (2)	C14—C13—C12	116.7 (3)
C9—N7—N6	108.1 (2)	C14—C13—H13	121.7
C10—N8—N9	107.5 (2)	C12—C13—H13	121.7
C11—N9—N8	106.4 (2)	C13—C14—C15	120.7 (3)
C10—N10—C11	104.2 (2)	C13—C14—H14	119.7
C10—N10—C12	128.4 (2)	C15—C14—H14	119.7
C11—N10—C12	127.3 (2)	C14—C15—C16	117.0 (3)
C16—N11—C12	116.5 (2)	C14—C15—H15	121.5
C18—N12—C17	104.4 (2)	C16—C15—H15	121.5
C18—N12—C16	128.9 (2)	N11—C16—C15	124.5 (2)
C17—N12—C16	126.7 (2)	N11—C16—N12	113.9 (2)
C17—N13—N14	106.9 (2)	C15—C16—N12	121.6 (2)
C18—N14—N13	107.2 (2)	N13—C17—N12	110.8 (2)
C19—N15—N16	106.8 (2)	N13—C17—H17	124.6
C20—N16—N15	107.0 (2)	N12—C17—H17	124.6
C19—N17—C20	103.8 (2)	N14—C18—N12	110.7 (2)
C19—N17—C21	128.2 (2)	N14—C18—H18	124.6
C20—N17—C21	128.0 (2)	N12—C18—H18	124.6
C21—N18—C25	115.9 (2)	N15—C19—N17	111.4 (2)
C27—N19—C26	103.8 (2)	N15—C19—H19	124.3
C27—N19—C25	128.9 (2)	N17—C19—H19	124.3
C26—N19—C25	127.3 (2)	N16—C20—N17	111.2 (2)
C26—N20—N21	106.1 (2)	N16—C20—H20	124.4
C27—N21—N20	107.6 (2)	N17—C20—H20	124.4
N1—C1—N3	111.1 (2)	N18—C21—C22	125.5 (2)
N1—C1—H1	124.4	N18—C21—N17	113.7 (2)
N3—C1—H1	124.4	C22—C21—N17	120.9 (2)
N2—C2—N3	111.2 (2)	C21—C22—C23	116.6 (3)
N2—C2—H2	124.4	C21—C22—H22	121.7
N3—C2—H2	124.4	C23—C22—H22	121.7
N4—C3—C4	124.6 (2)	C24—C23—C22	120.5 (3)
N4—C3—N3	114.1 (2)	C24—C23—H23	119.8
C4—C3—N3	121.3 (2)	C22—C23—H23	119.8
C3—C4—C5	116.8 (3)	C25—C24—C23	117.0 (2)
C3—C4—H4	121.6	C25—C24—H24	121.5
C5—C4—H4	121.6	C23—C24—H24	121.5
C6—C5—C4	120.6 (3)	N18—C25—C24	124.6 (2)
C6—C5—H5	119.7	N18—C25—N19	113.9 (2)
C4—C5—H5	119.7	C24—C25—N19	121.6 (2)
C5—C6—C7	117.1 (3)	N20—C26—N19	111.6 (3)
C5—C6—H6	121.4	N20—C26—H26	124.2
C7—C6—H6	121.4	N19—C26—H26	124.2
N4—C7—C6	124.5 (2)	N21—C27—N19	111.0 (3)
N4—C7—N5	114.0 (2)	N21—C27—H27	124.5
C6—C7—N5	121.5 (2)	N19—C27—H27	124.5
			
C1—N1—N2—C2	−0.4 (3)	N11—C12—C13—C14	0.0 (5)
C8—N6—N7—C9	0.0 (4)	N10—C12—C13—C14	−179.9 (3)
C10—N8—N9—C11	−0.2 (3)	C12—C13—C14—C15	−0.5 (5)
C17—N13—N14—C18	0.0 (3)	C13—C14—C15—C16	1.2 (5)
C19—N15—N16—C20	0.2 (3)	C12—N11—C16—C15	1.1 (4)
C26—N20—N21—C27	0.4 (4)	C12—N11—C16—N12	179.6 (2)
N2—N1—C1—N3	−0.3 (3)	C14—C15—C16—N11	−1.6 (5)
C2—N3—C1—N1	0.8 (3)	C14—C15—C16—N12	180.0 (3)
C3—N3—C1—N1	−178.3 (3)	C18—N12—C16—N11	−171.5 (3)
N1—N2—C2—N3	0.9 (3)	C17—N12—C16—N11	7.8 (4)
C1—N3—C2—N2	−1.1 (3)	C18—N12—C16—C15	7.1 (5)
C3—N3—C2—N2	178.1 (3)	C17—N12—C16—C15	−173.7 (3)
C7—N4—C3—C4	0.4 (4)	N14—N13—C17—N12	0.0 (3)
C7—N4—C3—N3	−179.3 (2)	C18—N12—C17—N13	0.0 (3)
C1—N3—C3—N4	−176.2 (3)	C16—N12—C17—N13	−179.4 (3)
C2—N3—C3—N4	4.9 (4)	N13—N14—C18—N12	0.0 (3)
C1—N3—C3—C4	4.2 (5)	C17—N12—C18—N14	0.0 (3)
C2—N3—C3—C4	−174.7 (3)	C16—N12—C18—N14	179.4 (3)
N4—C3—C4—C5	−0.1 (5)	N16—N15—C19—N17	−0.3 (3)
N3—C3—C4—C5	179.5 (3)	C20—N17—C19—N15	0.3 (3)
C3—C4—C5—C6	0.1 (5)	C21—N17—C19—N15	−179.8 (3)
C4—C5—C6—C7	−0.4 (5)	N15—N16—C20—N17	0.0 (3)
C3—N4—C7—C6	−0.7 (4)	C19—N17—C20—N16	−0.2 (3)
C3—N4—C7—N5	179.1 (2)	C21—N17—C20—N16	180.0 (3)
C5—C6—C7—N4	0.7 (5)	C25—N18—C21—C22	−0.2 (4)
C5—C6—C7—N5	−179.1 (3)	C25—N18—C21—N17	179.6 (2)
C9—N5—C7—N4	−178.5 (3)	C19—N17—C21—N18	173.2 (3)
C8—N5—C7—N4	0.6 (4)	C20—N17—C21—N18	−7.0 (4)
C9—N5—C7—C6	1.3 (5)	C19—N17—C21—C22	−7.0 (5)
C8—N5—C7—C6	−179.6 (3)	C20—N17—C21—C22	172.9 (3)
N7—N6—C8—N5	0.2 (4)	N18—C21—C22—C23	0.2 (5)
C9—N5—C8—N6	−0.4 (4)	N17—C21—C22—C23	−179.6 (3)
C7—N5—C8—N6	−179.7 (3)	C21—C22—C23—C24	−0.1 (5)
N6—N7—C9—N5	−0.2 (4)	C22—C23—C24—C25	0.0 (5)
C8—N5—C9—N7	0.4 (4)	C21—N18—C25—C24	0.1 (4)
C7—N5—C9—N7	179.7 (3)	C21—N18—C25—N19	179.3 (2)
N9—N8—C10—N10	0.5 (4)	C23—C24—C25—N18	−0.1 (5)
C11—N10—C10—N8	−0.5 (3)	C23—C24—C25—N19	−179.1 (3)
C12—N10—C10—N8	−179.7 (3)	C27—N19—C25—N18	−175.5 (3)
N8—N9—C11—N10	0.0 (3)	C26—N19—C25—N18	1.5 (4)
C10—N10—C11—N9	0.3 (3)	C27—N19—C25—C24	3.7 (5)
C12—N10—C11—N9	179.5 (3)	C26—N19—C25—C24	−179.3 (3)
C16—N11—C12—C13	−0.3 (4)	N21—N20—C26—N19	−0.5 (4)
C16—N11—C12—N10	179.6 (2)	C27—N19—C26—N20	0.4 (4)
C10—N10—C12—N11	173.0 (3)	C25—N19—C26—N20	−177.2 (3)
C11—N10—C12—N11	−6.0 (4)	N20—N21—C27—N19	−0.1 (4)
C10—N10—C12—C13	−7.1 (5)	C26—N19—C27—N21	−0.1 (4)
C11—N10—C12—C13	173.8 (3)	C25—N19—C27—N21	177.4 (3)

### Hydrogen-bond geometry (Å, °)

**Table:**

*D*—H···*A*	*D*—H	H···*A*	*D*···*A*	*D*—H···*A*
O6—H6A···N21^i^	0.86 (1)	1.95 (1)	2.801 (3)	172 (3)
O6—H6B···N1^ii^	0.86 (1)	2.04 (1)	2.878 (3)	166 (3)
O5—H5A···O6	0.87 (1)	1.86 (2)	2.707 (3)	166 (4)
O5—H5B···O1^iii^	0.86 (1)	2.01 (1)	2.864 (4)	171 (4)
O4—H4A···N14^iv^	0.86 (1)	1.95 (1)	2.808 (3)	173 (4)
O4—H4B···N8^v^	0.86 (1)	2.09 (2)	2.907 (3)	160 (3)
O3—H3B···O5	0.86 (1)	2.03 (2)	2.868 (4)	166 (4)
O3—H3A···O4	0.85 (1)	2.01 (1)	2.855 (3)	173 (3)
O2—H2B···O4	0.86 (1)	2.00 (1)	2.851 (4)	172 (4)
O2—H2A···O1	0.87 (1)	2.03 (1)	2.886 (3)	171 (3)
O1—H1B···N15^vi^	0.86 (1)	2.05 (2)	2.861 (3)	158 (3)
O1—H1A···N7	0.86 (1)	1.97 (1)	2.820 (3)	171 (3)

Symmetry codes: (i) *x*−1, *y*, *z*; (ii) −*x*, *y*+1/2, −*z*+3/2; (iii) *x*, −*y*+3/2, *z*−1/2; (iv) −*x*+2, *y*−1/2, −*z*+3/2; (v) *x*+1, *y*, *z*; (vi) −*x*+1, *y*+1/2, −*z*+3/2.
